# Diagnostic Application of Fluorine-18 Fluorodeoxyglucose Positron Emission Tomography/Computed Tomography in the Treatment of Oral Squamous Cell Carcinoma in an African Pygmy Hedgehog

**DOI:** 10.3390/ani14243679

**Published:** 2024-12-20

**Authors:** Jaegyeong Shin, Nari Kim, Gwi-Ho Jeon, Taesik Yun, Byeong-Teck Kang, Dong-Hyuk Jeong

**Affiliations:** 1 College of Veterinary Medicine, Chungbuk National University, Cheongju 28644, Republic of Korea; shinjk8646@naver.com (J.S.); queng91@gmail.com (N.K.); fermium@cbnu.ac.kr (T.Y.); kangbt@cbnu.ac.kr (B.-T.K.); 2 Su Veterinary Clinic, Cheongju 28665, Republic of Korea; vet9530@naver.com; 3 Wildlife Center of Chungbuk, Cheongju 28116, Republic of Korea

**Keywords:** African pygmy hedgehog, *Atelerix albiventris*, oral squamous cell carcinoma, 18F-FDG PET/CT, metastasis assessment

## Abstract

Neoplastic diseases are common in African pygmy hedgehogs (*Atelerix albiventris*), with squamous cell carcinoma (SCC) being the most prevalent oral tumor. Traditional imaging techniques like computed tomography (CT) have limitations in accurately defining tumor extent and detecting metastasis. In this study, a hedgehog with a suspected oral tumor underwent 18F-FDG PET/CT imaging, revealing a hypermetabolic lesion consistent with SCC and indications of metastatic activity. The lesion was too extensive for a lumpectomy, and suspected metastasis led to the decision for euthanasia due to the impracticability of further treatment. A histopathological examination after autopsy confirmed the diagnosis and metastatic spread, as suggested by PET/CT findings. This is the first case report demonstrating the potential use of 18F-FDG PET/CT in hedgehogs, facilitating the accurate staging and management of oral SCC. Further research is needed to establish PET/CT as a standard diagnostic tool for neoplastic diseases in hedgehogs.

## Simple Summary

This case report evaluates the effectiveness of Fluorine-18 fluorodeoxyglucose positron emission tomography/computed tomography (18F-FDG PET/CT) in diagnosing tumor malignancy and assessing metastasis in an African pygmy hedgehog (*Atelerix albiventris*) with oral squamous cell carcinoma. Our findings suggest that 18F-FDG PET/CT can be a useful tool for assessing malignancy and metastasis in tumors in African pygmy hedgehogs.

## 1. Introduction

African pygmy hedgehogs (*Atelerix albiventris*) commonly develop neoplastic diseases, with retrospective studies indicating a prevalence ranging from 29% to 53% during necropsies [1,2,3]. These neoplasms are predominantly malignant (up to 85%) and often lead to a poor prognosis [1]. Among hedgehogs with oral neoplastic disease, squamous cell carcinoma (SCC) is the most prevalent type of neoplasm [1,4,5]. SCC metastasizes slowly, with pulmonary metastases occurring in a few cases [6]. Other neoplastic cases include peripheral odontogenic fibroma [7], osteoma [8], osteochondroma [9], osteosarcoma [4,8], and fibrosarcoma [1,10].

The spectrum of oral lesions typically observed in hedgehogs include both neoplastic and non-neoplastic growth [1,5,11]. A retrospective study on privately owned hedgehogs identified twenty-seven oral cavity masses, eight of which were non-neoplastic (gingival hyperplasia), and nineteen neoplastic (seventeen SCCs and two mesenchymal tumors) [11]. This study states that histopathology is essential for a definitive diagnosis, although clinical signs, growth patterns, and anatomic location can aid in identifying SCC.

Treatment options for hedgehogs with SCC include chemotherapy [12], electrochemotherapy (ECT) [13], and lumpectomy [14], albeit with limited success due to metastasis. Computed tomography (CT) remains the primary tool for treatment planning; however, its limitations in accurately defining tumor extent and detecting metastasis are acknowledged [15].

In response to these limitations, positron emission tomography/computed tomography (PET/CT) has gained traction for head and neck cancer staging in humans [16] and cats [15,17], offering superior sensitivity for detecting cancer infiltration. PET/CT is a powerful tool for assessing metastatic disease, providing detailed metabolic and anatomical insights that aid in the precise staging and management of cancer [18].

The authors found no existing reports to date on the use of PET/CT for tumors in hedgehogs. Therefore, this case report evaluates the effectiveness of PET/CT in diagnosing tumor malignancy and evaluating tumor metastasis in hedgehogs with oral neoplastic diseases.

## 2. Case Description

A client-owned, 3-year-old male African pygmy hedgehog was first seen by a primary care veterinarian at Su Veterinary Clinic in July 2023 due to hyporexia. After the patient was anesthetized, a physical examination revealed that both the left mandibular and maxillary teeth were covered by a swollen, tumor-like gingiva. The patient was treated with prednisolone (PDS) (1 mg/kg orally (PO)), enrofloxacin (5 mg/kg PO), and famotidine (0.5 mg/kg PO) twice daily for 7 days.

A biopsy was performed on the damaged teeth and swollen gingiva to collect samples for histopathological examination. One sample from the teeth and five samples of gingival tissue, with depths ranging from 0.7 mm to 2.7 mm, were analyzed. Histopathological analysis revealed severe gingivitis and proliferative findings in the mucosal epithelial cells of the gingiva. The proliferative epithelial cells displayed bridge formations, proliferating in a cord-like growth pattern or forming islands. Keratin pearls and mitotic figures were not observed in this shallow sample. Based on these findings, acanthomatous ameloblastoma was diagnosed.

The oral mass in the gingival area continued to spread despite treatment and was subsequently treated with oclacitinib (0.6 mg/kg PO) for one day, along with PDS (1 mg/kg PO), clindamycin (5 mg/kg PO), and famotidine (0.5 mg/kg PO) for 7 days. All medications were administered twice daily. After 7 days, the oral mass appeared to be stable. Doxycycline (5 mg/kg PO) was added to the previous treatment regimen, administered twice daily for another 7 days, to prevent secondary infection. However, the mass began to spread again 29 days after the initial treatment began, prompting a switch from oclacitinib to toceranib (2.5 mg/kg PO) twice daily for one day. After two weeks, an abscess formed at the site of the tooth root, among the tumor sites, with a cheese-like consistency. As no improvement was observed, the patient was referred to Chungbuk National University Veterinary Teaching Hospital (CBNU-VTH) in October.

The patient had an overall swollen left side of the face and was unable to open his eyes. Forced breathing was confirmed, and an X-ray scan revealed gas accumulation in the intestines. After collecting blood samples from the cranial vena cava, a complete blood cell count (CBC) was performed using the ProCyte Dx (IDEXX, Westbrook, ME, USA) and blood chemistry tests were conducted using the VETSCAN VS2 (Zoetis, Parsippany, NJ, USA). The CBC results showed inflammation signs with increased neutrophil and monocyte counts. The blood chemistry results indicated impaired kidney and liver function, as evidenced by elevated levels of alanine transaminase, alkaline phosphatase, calcium, blood urea nitrogen, and globulin. The patient’s blood glucose levels were very low, necessitating oral glucose administration. Due to persistent severe pain responses, butorphanol was administered subcutaneously at a dosage of 0.1 mg/kg.

**Figure 1:**
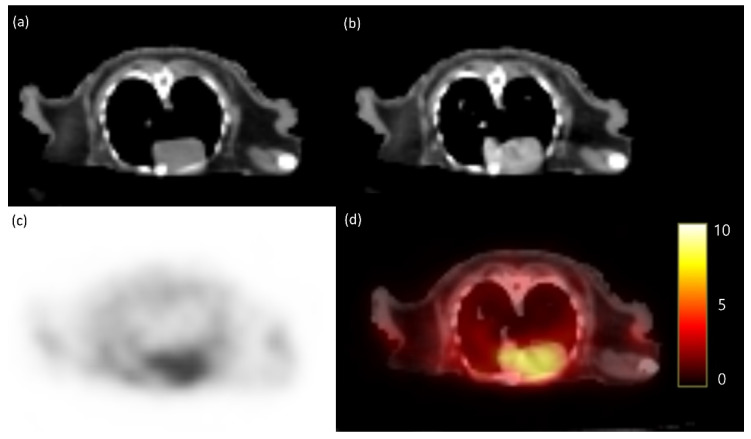
FDG-PET findings in a hedgehog (*Atelerix albiventris*) in the pulmonary region. Pre-contrast CT image (**a**), post-contrast CT image (**b**), FDG-PET (**c**), and PET/CT fusion (**d**) images. Pre-contrast CT image (**a**) showing anatomical structures without enhancement. Post-contrast CT image (**b**) demonstrating enhanced anatomical structures. FDG-PET image (**c**), where low and high FDG uptakes are represented by blackish and whitish colors. Fused FDG-PET/CT image (**d**) using a hot metal color scale, where low and high FDG uptakes are represented by blackish-to-reddish and yellowish colors. CT, computed tomography; FDG, 18F-fluorodeoxyglucose; PET, positron emission tomography.

The PET/CT images in this study were acquired using the Discovery STE PET/CT system (General Electric Medical Systems, Waukesha, WI, USA). The patient was anesthetized with isoflurane using a mask and positioned in dorsal recumbency with the head immobilized to ensure accurate imaging. Following the induction of anesthesia, 0.17 mCi/kg of fluorodeoxyglucose (FDG) was administered intravenously via the cephalic vein. Low-dose CT images were acquired, followed by static PET images obtained 60 min post-injection. Whole-body PET images were acquired every 5 min per bed position. The PET image analysis was performed using OsiriX MD v10.0 (Pixmeo Sarl, Geneva, Switzerland). The regions of interest (ROIs) were manually delineated on the PET/CT fusion images. Metabolic activity of the ROIs was converted to a standardized uptake value (SUV) as follows: SUV = average tissue concentration of FDG (MBq/mL)/injected dose (MBq) per body weight (g). This resulted in a SUVmax of 10.31 and an SUVmean of 8.73 in the maxillary region. Furthermore, an SUVmax of 7.03 and SUVmean of 5.30 were observed in the left side of the lung (Figure 1). The lesion was deemed too extensive for lumpectomy, and metastasis to other areas, including the left lung, was suspected. Due to the impracticability of further treatment, euthanasia was chosen with the consent of the owner of the patient.

**Figure 2:**
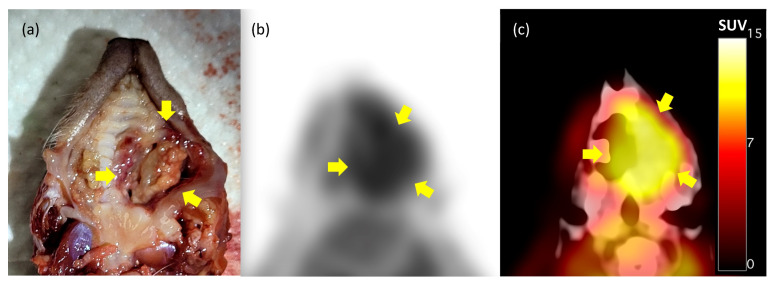
Necropsy and FDG-PET findings in a hedgehog (*Atelerix albiventris*) with an oral tumor. (**a**) An oral mass (arrows) found in the left maxillary region. FDG-PET (**b**) and PET/CT fusion (**c**) images. High FDG uptake is observed in the tumor lesion (arrows), and the maximal SUV of the lesion is 10.31.

Following euthanasia, a post-mortem examination was performed. The lesion site of the maxillary bone tumor corresponded to the area of high FDG uptake on the PET/CT (Figure 2). Additional PET/CT fused images from multiple planes (coronal, sagittal, and axial) of the maxillary regions were obtained to illustrate tracer uptake and distribution in the head (Supplementary Figure S1). Several other lesions were observed in the left eye, lungs, and kidneys. Samples were collected for histopathological analysis, including a cross-section of the oral tissue and skull where the protruding mass was most clearly visible, as well as ocular tissue and six cross-sections of the left lung.

**Figure 3:**
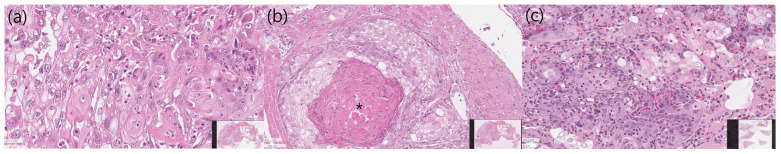
Photomicrographs of histopathological evaluation in oral (**a**,**b**) and lung (**c**) tissue of a hedgehog (*Atelerix albiventris*) with SCC. (**a**) Oral tissue: tumorous epithelial proliferation with nuclear pleomorphism and mitotic figures. Hematoxylin and eosin (H&E) stain. Scale bar = 50 μm. (**b**) Oral tissue: stratified squamous epithelial cells with a keratin pearl (asterisk). H&E stain. Scale bar = 100 μm. (**c**) Lung tissue: pulmonary edema and metastatic squamous cell carcinoma with inflammatory cell infiltration. H&E stain. Scale bar = 50 μm.

The histological examination revealed a tumorous proliferation of epithelial cells in the oral tissue, characterized by marked cellular and nuclear pleomorphism, with 0–4 mitotic figures per high-power field (Figure 3a). The tumor cells aggressively infiltrated the inflamed muscular and connective tissues. They predominantly exhibited a stratified squamous epithelial cell morphology in an islet pattern, with the central formation of keratin pearls (Figure 3b).

In the skull tissue, there was evident tumorous proliferation of epithelial cells similar to that observed at the primary site, along with pronounced cellular and nuclear pleomorphism. In the left ocular tissue, features consistent with SCC, nearly identical to the primary tumor, were observed in both the mass attached posteriorly to the retino-scleral complex and the mass adjacent to the optic disk. The tumors were growing inwards towards the inner part of the eye.

A histological examination of the left lung tissue revealed pulmonary edema and metastatic SCC (Figure 3c). Multifocal micronodular structures attributable to SCC were identified, showing histological features consistent with those of the primary tumors. Cells exhibiting a bizarre morphology with more than four nucleoli were observed. Furthermore, the infiltration of various inflammatory cells, including eosinophils, was observed within the tumor mass.

## 3. Discussion

To the best of our knowledge, this is the first case report to evaluate malignancy and metastasis to other organs in hedgehogs using PET/CT imaging. The tumor was assessed through a post-mortem histopathological examination to verify its consistency with the PET/CT results.

FDG-PET leverages the characteristic higher glucose metabolism of malignant tumors compared to that of normal tissues, making it a useful tool for cancer diagnosis, staging, and restaging [19]. Hybrid PET/CT enhances the diagnostic accuracy of FDG-PET and CT in cancer patients, influencing both diagnostic and therapeutic decisions in patient management [20]. This combined imaging enables a more accurate assessment of the location and metastasis of malignant tumors.

We investigated whether metabolic activity indicators can accurately assess the location and metastasis of oral squamous cell carcinoma (OSCC) in hedgehogs and whether this method could be applied. Due to their smaller organs compared with those of humans and cats, it was uncertain if tumor and metastasis evaluation using PET/CT imaging would be feasible. However, PET/CT imaging revealed severe malignancy of the oral tumor and detected metastasis in the left lung. The SUVmax and SUVmean values in the oral region were 10.31 and 8.33, respectively. In cats, previous records indicate that the SUVmax value in the oral region was 9.88, and the SUVmean value was 5.39 [15]. These similar values suggest the potential applicability of PET/CT for evaluating OSCC in hedgehogs. The PET/CT results indicated significant metabolic activity in the maxillary region, suggesting an aggressive lesion. Axial views also demonstrated tumor infiltration and turbinate bone lysis in the left nasal cavity. In the left lung, the SUVmax value was 7.03, and the SUVmean value was 5.30. No comparison with lung SUV values in PET/CT-imaged cats with OSCC was possible, as there were no lung metastasis cases reported; however, metastasis was inferred due to the high uptake in the lung area.

Consistent with the PET/CT imaging results, the post-mortem examination confirmed highly malignant masses in the oral cavity and lungs. Histopathological examination verified metastasis in the oral cavity, skull, eyes, and lungs. Before the patient was referred to CBNU-VTH, a superficial biopsy identified the tumor as being benign, diagnosing it as acanthomatous ameloblastoma. However, PET/CT imaging indicated malignancy, and a subsequent deeper biopsy confirmed the diagnosis of OSCC. This indicates that, without excising the tumor to its full depth, there is a risk of misdiagnosing SCC as another neoplasm. PET/CT can help prevent such misdiagnoses by assessing malignancy and evaluating metastasis.

The current best treatment methods for OSCC include lumpectomy, chemotherapy, and ECT, with metastasis evaluation being essential for accurate and efficient treatment planning. Even with successful surgery, the prognosis may be poor without metastasis evaluation, leading to postoperative mortality [1]. In this case, PET/CT imaging revealed that the lesion was too large for a lumpectomy, and metastasis in the lung was confirmed, rendering further treatment impossible. If an early diagnosis is made through PET/CT imaging in patients with an oral mass, more effective treatment could be achieved based on the evaluation of the malignancy and metastasis of the tumor.

A detailed evaluation of metastases in the oral cavity, nasal cavity, and eyes was impossible due to the reduced PET/CT resolution resulting from the small size of the hedgehog. This limitation allowed only for an approximate evaluation of metastasis in the other organs. However, this report suggests the potential use of PET/CT for assessing malignancy and metastasis in tumors in hedgehogs, including SCC.

## 4. Conclusions

This case report highlights the potential utilization of 18F-FDG PET/CT imaging in diagnosing and staging malignant tumors, specifically OSCC, in African pygmy hedgehogs (*Atelerix albiventris*). PET/CT imaging provided detailed metabolic and anatomical insights, revealing the severity of malignancy of the oral tumor and detecting lung metastasis. Despite the resolution limitations of FDG uptake inherent to using human-sized PET/CT system for a small hedgehog, the imaging effectively identified the extent of the primary tumor and its metastases. These findings were confirmed through a post-mortem histopathological examination. This suggests that PET/CT imaging could play a crucial role in early diagnosis, accurate staging, and treatment planning for neoplastic diseases in hedgehogs, potentially improving clinical outcomes. Further research is needed to establish PET/CT as a standard diagnostic tool for tumors in hedgehogs, and to refine imaging techniques to allow for a better resolution and accuracy in small animals.

## Data Availability

The data presented in this study are available on request from the corresponding author.

## Supplementary Materials

The following supporting information can be downloaded at: https://www.mdpi.com/article/10.3390/ani14243679/s1, Figure S1: FDG-PET/CT images in a hedgehog (*Atelerix albiventris*) showing tracer uptake in the maxillary region. Coronal (**a**), Sagittal (**b**), and axial views (**c**–**f**) of FDG PET/CT fused images. Coronal view of the fused FDG–PET/CT image (**a**) demonstrating increased FDG uptake at the left maxillary region. Sagittal view of the fused FDG–PET/CT image (**b**), with dashed lines indicating the axial cross-section levels shown in (**c**–**f**). Axial views of fused FDG–PET/CT images at different levels (**c**–**f**), highlighting tumor infiltration and turbinate bone lysis (arrows) in the left nasal cavity.
