# Supplementary material for: Diagnostic Application of Fluorine-18 Fluorodeoxyglucose Positron Emission Tomography/Computed Tomography in the Treatment of Oral Squamous Cell Carcinoma in an African Pygmy Hedgehog
Source: Animals (Basel). 2024 Dec 20;14(24):3679. doi: 10.3390/ani14243679 (PMC11672570; doi:10.3390/ani14243679)
Supplement: Supplementary file 1 [file animals-14-03679-s001.zip › animals-3325943-supplementary.pdf]

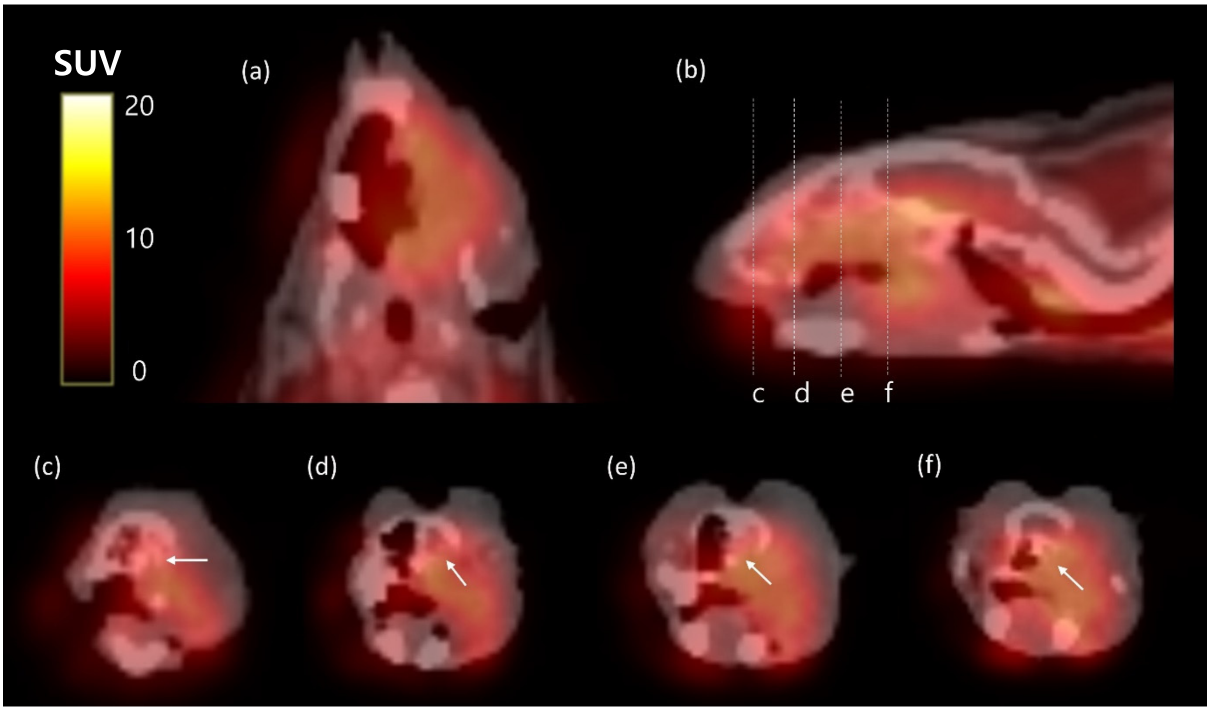

Supplementary Figure S1. FDG-PET/CT images in a hedgehog (*Atelerix albiventris*) showing tracer uptake in the maxillary region. Coronal (**a**), Sagittal (**b**), and axial views (**c**–**f**) of FDG PET/CT fused images. Coronal view of the fused FDG–PET/CT image (**a**) demonstrating increased FDG uptake at the left maxillary region. Sagittal view of the fused FDG–PET/CT image (**b**), with dashed lines indicating the axial cross-section levels shown in (**c**–**f**). Axial views of fused FDG–PET/CT images at different levels (**c**–**f**), highlighting tumor infiltration and turbinate bone lysis (arrows) in the left nasal cavity.
